# Ice phenology dataset reconstructed from remote sensing and modelling for lakes over the Tibetan Plateau

**DOI:** 10.1038/s41597-022-01863-9

**Published:** 2022-12-02

**Authors:** Yanhong Wu, Linan Guo, Bing Zhang, Hongxing Zheng, Lanxin Fan, Haojing Chi, Junsheng Li, Shenglei Wang

**Affiliations:** 1 International Research Centre of Big Data for Sustainable Development Goals, Beijing, 100094 China; 2 Key Laboratory of Remote Sensing Science, Aerospace Information Research Institute, Chinese Academy of Sciences, Beijing, 100094 China; 3 Institute of Tibetan Plateau Research, Chinese Academy of Sciences, Beijing, 100101 China; 4 University of the Chinese Academy of Sciences, Beijing, 100049 China; 5 CSIRO Land and Water, Canberra, ACT 2601 Australia

**Keywords:** Limnology, Hydrology

## Abstract

The Tibetan Plateau (TP) is a region sensitive to global climate change and has been experiencing substantial environmental changes in the past decades. Lake ice phenology (LIP) is a perceptible indicator reflecting changes of lake thermodynamics in response to global warming. Lake ice phenology over the Tibetan Plateau is however rarely observed and recorded. This research presents a dataset containing 39-year (1978–2016) lake ice phenology data of 132 lakes (each with area >40 km^2^) over the Tibetan Plateau by combining the strengths of both remote sensing (MOD11A2, MOD10A1) and numerical modelling (air2water). Data validation shows that the ice phenology data derived by our method is highly consistent with that based on existing approaches (with R^2^ > 0.75 for all phenology index and RMSE < 5d). The dataset is valuable to investigate the lake-atmosphere interactions and long-term hydrothermal change of lakes across the Tibetan Plateau.

## Abstract (summary)

**Table:** 

Measurement(s)	lake ice phenology
Technology Type(s)	remote sensing and numerical modeling

## Background & Summary

The formation and duration of ice cover for lakes in the Cryosphere plays an important role in thermodynamics of lakes. It interacts with lake water temperature and shapes aquatic ecosystems as well. Lake ice phenology (LIP, e.g., dates of freezing-up and breaking-up) are closely related to temperature variation^1,2^. Change of lake ice phenology in the Cryosphere is considered as one of the most reliable pieces of evidence reflecting global warming^3–5^. It could subsequently lead to substantial alteration in biogeochemical processes (e.g., influencing growth of plankton and causing anoxia in deep water^6^). Research efforts on investigating changes of lake ice phenology are mainly based on ground observation, satellite observation or modelling^7–10^. Most of the research, however, are for lakes locating in North America or Northern Europe as there are relatively abundant in ground observations^10^.

Lake ice phenology observations are not always widely available particularly for regions like the Tibetan Plateau (TP), where ground-based observation is challenging and costly. Observation from space therefore has been becoming more attractive in investigating the lake ice phenology. For instance, with stratified random sample from Sentinel-2 and PlanetScope, Pickens *et al*. (2021) reported that 41% of inland water area around the globe were seasonally frozen^11^. Though with uncertainties^12^ from both data sources^13–15^ and extraction methods, various satellite-based observations have shown their merits in tracking the freezing-thawing cycle of lake ice cover^16^. The most commonly used satellite data includes that from the optical sensors like Advanced Very High Resolution Radiometer (AVHRR)^17^ and Moderate-resolution Imaging Spectroradiometer (MODIS)^18^, the passive microwave sensors like Scanning Multichannel Microwave Radiometer (SMMR)^3^, the Advanced Microwave Scanning Radiometer for EOS (AMSR-E)^19^ and Special Sensor Microwave/Imager (SSM/I)^20^. Active microwave also shows capabilities in monitoring lake ice status based on sensors like European Remote Sensing Satellite (ERS)-1/2 synthetic aperture radar (SAR)^21^ and Radarsat-1/2 SAR^22^, but the narrow swath width and the relatively low temporal resolution limited the application of the technology to monitor lake ice phenology at a daily scale and for a large area. It is also noted that the satellite-based observations of lake ice phenology mostly start from the late 1990s and could be with considerable data gaps. For example, the currently available dataset for lake ice phenology in the TP is that derived from microwave brightness temperature measured by AMSR-E, the Advanced Microwave Scanning Radiometer 2 (AMSR2) and Micro-Wave Radiation Imager (MWRI). The dataset however is limited to 51 lakes on the TP covering the period from 2002 to 2015^23^.

In the literature of limnology, numerous research efforts also have been invested on developing mathematical models to reconstruct lake ice phenology for the historical period or to predict the response of lake ice phenology to a warming climate^5,24^. The model-based approach goes beyond reproducing data on lake ice phenology and can be used to quantify the response of lake ice phenology to climate change or climate variation. The models may cover different level of details on lake thermodynamics, subject to their research focuses^25–29^. It is recognized that a well simplified process-based model (like air2water^30,31^) could perform as good as a more complicated model (like MINLAKE^26^, LIMNOS^27^, HIGHTSI^28^) if the research interest is mainly on the timing of freezing and thawing of lake ice. The simplified model could be more competitive and applicable for regions with little *in-situ* observations (like the TP) as it requires much less data for running and calibration.

**Fig. 1:**
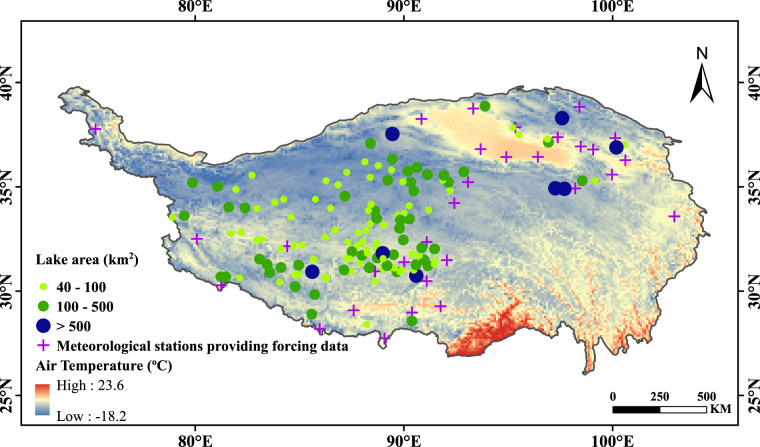
Locations of studied lakes in the Tibetan Plateau.

The dataset herein provides complete, consistent and continuous time series (1978–2017) of reconstructed ice phenology for 132 lakes in the TP (Fig. 1) by combing the strengths of remote sensing and mathematical modelling forced by meteorological data. The lake surface water temperature (LSWT) derived from MODIS products (MOD11A2)^32^ is applied to calibrate and validate the numerical model in reproducing the consecutive daily surface water temperature for the studied period, based on which algorithm has been developed to determine the ice phenology indices (including dates of freezing-up and breaking-up and durations). Lake ice phenology derived directly from satellite observations in this research and by other researchers is used to validate the reconstructed LIP^33^.

The reconstructed LIP represents a unique dataset to investigate the long-term trends of lake ice phenology in the TP under a warming climate. The dataset can also be used for a variety of applications related to climate change, limnology, hydrology, and aquatic ecology. It is conductive to assess the impacts of climate warming on dynamics of water/heat budget and aquatic biota in lakes across the TP.

## Methods

**Fig. 2:**
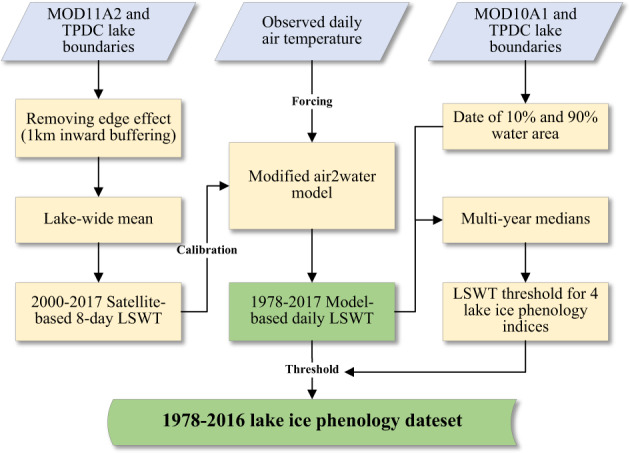
Flowchart in producing the lake ice phenology (LIP) dataset.

Figure 2 shows the general framework of our study in determining the lake ice phenology for the 132 largest lakes across the Tibetan Plateau. The satellite-based lake surface temperature is used to calibrate the modified air2water model. The air temperature data from meteorological station is the solely required input data to drive the model. The temperature threshold of lake ice phenology is determined with the aid of MODIS snow cover product. Details of the procedures and methods are described in the following sections.

### Data

The modified air2water model requires solely air temperature as input. For the TP, the best publicly accessible ground-based observation of daily air temperature is that from the dataset of daily climate data from Chinese surface stations (V3.0) provided by China Meteorological Data Service Centre of National Meteorological Information Centre (http://data.cma.cn/data/). The daily air temperature from the dataset is only available at 31 meteorological stations located in the TP. The spread and density of the meteorological stations are big constraints for earth system research in the region. We noticed that quite a few research efforts have been invested to provide reliable reanalysis climate datasets for the TP (e.g., China meteorological forcing dataset). The public available climate reanalysis datasets however are found not necessarily of high good quality for our simulation when we compare them against the observations from the meteorological stations. Hence, we use the air temperature interpolated from the nearest station with altitude adjustment (by a lapse rate of 0.65 °C 100 m^−1^)^34^ to provide air temperature input to drive the modified air2water model.

The direct output of the model is lake surface water temperature. Hence, the model is firstly calibrated and validated against the satellite-based observation of lake surface water temperature deriving from the MOD11A2^32^ (i.e., Terra product of Land Surface Temperature/Emissivity 8-day L3 Global 1 km). The surface water temperature of a specific lake is the lake-wide mean temperature based on all pixels within the lake. Boundaries of the lakes (shapefiles included in the dataset) are mainly from National Tibetan Plateau Data Center (TPDC, http://data.tpdc.ac.cn) and are cross-checked by maps from HydroLAKES (http://www.hydrosheds.org/)^35^ and Google Earth. The lake-wide MODIS-based surface water temperature is the mean of daytime and night-time LST in the MOD11A2. All the data processing procedures are implemented via Google Earth Engine (GEE)^36^.

Prior to determine the lake ice phenology given the modelled lake surface temperature, temperature thresholds should be defined, which is done herein with the aid of remotely sensed snow cover product, i.e., MOD10A1^37^. The MOD10A1 dataset is a snow cover product with a spatial resolution of 500 m containing daily, gridded snow cover and albedo derived from radiance data acquired by MODIS on board the Terra satellite. Snow cover is identified using the Normalized Difference Snow Index (NDSI) and a series of screens designed to alleviate errors and flag uncertain snow cover detections^37^.

### Lake surface temperature modeling

We used a slightly modified air2water model to simulate the daily lake surface temperature for the period 1978–2017. The air2water^27,28^ is a semi-physical model designed to simulate lake surface temperature principally based on lake surface energy balance expressed as:

1 $$\rho {c}_{p}{V}_{s}\frac{d{T}_{w}}{dt}=A{H}_{net}$$

where *T*_*w*_ is water surface temperature at time *t* (day step in this study), *H*_*net*_ is the net heat flux per unit surface, *A* is the surface area of the lake, *V*_*s*_ is the volume of water involved in the heat exchange with the atmosphere, *ρ* and *c*_*p*_ is the density and specific heat capacity of water. The model considers all contributions in the energy balance (*H*_*net*_) as a function of air temperature or is included in the parameters of the model. The original air2water model has simplified the lake energy balance by introducing eight calibratable parameters a_1–8_ with air temperature as the only required input, which can be expressed as:

2 $$\frac{d{T}_{w}}{dt}=\frac{1}{\delta }\left\{{a}_{1}+{a}_{2}{T}_{a}-{a}_{3}{T}_{w}+{a}_{5}\,{\cos }\left[2\pi \left(\frac{t}{{t}_{y}}-{a}_{6}\right)\right]\right\}$$

3 $$\delta =\left\{\begin{array}{c}{\exp }\left(-\frac{{T}_{w}-{T}_{h}}{{a}_{4}}\right),\quad \quad \quad for\quad {T}_{w}\ge {T}_{h}\\ {\exp }\left(-\frac{{T}_{h}-{T}_{w}}{{a}_{7}}\right)+{\exp }\left(-\frac{{T}_{w}}{{a}_{8}}\right),\quad \quad for\quad {T}_{w}\le {T}_{h}\end{array}\right.$$

where, *T*_*a*_ is air temperature, *t*_*y*_ is days of a year. *δ* = *D/D*_*r*_ is normalized well-mixed depth, where *D* is the depth of well-mixed surface layer (the epilimnion thickness), *D*_*r*_ is the maximum thickness. *T*_*h*_ is the deep water temperature assumed to be 4 °C.

The model has been tested efficient in different regions^38,39^. However, the original air2water is limited to simulate the surface temperature of open water, which results in difficulties for applications in lakes with long ice cover duration. For this sake, we assume that when the lake is completely covered by ice, the heat exchange between air and water is blocked and surface energy balance would be replaced by land surface energy balance without mixing depth in water^36^, which indicates *δ* = 1 and Eq. (2) be rewritten as:

4 $$\frac{d{T}_{i}}{dt}={a}_{9}+{a}_{10}{T}_{a}-{a}_{11}{T}_{w}+{a}_{12}\,{\cos }\left[2\pi \left(\frac{t}{{t}_{y}}-{a}_{13}\right)\right]$$

where *T*_*i*_ is the ice surface temperature, *a*_9–13_ have similar physical significance to *a*_1,2,3,5,6_. To represent the shifts of lake between open water and ice-covered, two additional parameters a_14_ and a_15_ are introduced to determine the states of the lake, which the lake surface water temperature (*LSWT*) can be expressed as:

5 $$LSWT=\left\{\begin{array}{c}{T}_{w},\;for\;{T}_{L}\ge {a}_{15}\\ {T}_{i},\;for\;{T}_{L}\le {a}_{14}\\ (1-{K}_{ice}){T}_{w}+{K}_{ice}{T}_{i},\;for\;{a}_{15} > {T}_{L} > {a}_{14}\end{array}\right.$$

where we set ${K}_{ice}=\sqrt{({a}_{15}-LSWT)/({a}_{15}-{a}_{14})}$ as the proportion of ice on the surface of the lake. Same as the original model, the model equations were solved numerically by using the Crank-Nicolson numerical scheme at daily time step.

The model is calibrated against the LSWT derived from MOD11A2 using the PSO (Particle Swarm Optimization) approach^31,40^. The objective function of model calibration is the root mean square error (RMSE). To ensure the rationality of the calibrated model parameters, a physically consistent priori range are assigned to each parameter^31^ and D_r_ is bounded by the average depth of the lake obtained from Records of Lakes in China^41^ and HydroLAKES^35^. The calibration period is 2000–2012, while 2013–2017 is for validation.

### Temperature thresholds for ice phenology

**Fig. 3:**
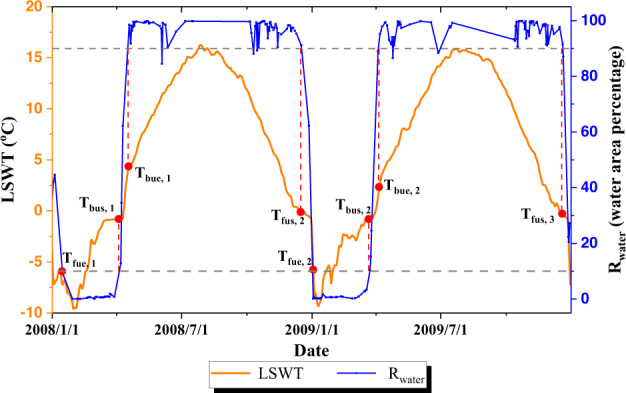
Determination of lake surface water temperature (LSWT) thresholds for identifying the lake ice phenology indicators. *T*_*FUS, i*_ *T*_*FUE,i*_, *T*_*BUS,i*_ and *T*_*BUE,i*_ are the temperature threshold cand***i***dates derived for each specific year *i* (=1,2,3). ***R***_***water***_ is the percentage of open water area within a lake. Data of this figure is from the Qinghai Lake for the period 2008–2009.

The lake surface temperature output from air2water model is used to derive from lake ice phenology given prior defined temperature thresholds. The temperature thresholds are defined with the aid of the snow cover products (MOD10A1), where the MOD10A1 images with cloud proportion over the lake exceeding 20% are neglected. For each lake, the ratio of “inland water” (*R*_*water*_) to total lake area is firstly calculated as: ${R}_{water}={N}_{water}/\left({N}_{water}+{N}_{ice}\right)$, where *N*_*water*_ is the pixel number of “inland water”, while *N*_*ice*_ is the pixel number of “ice”. The lake is assumed to be open when *R*_*water*_ > 90% and frozen when *R*_*water*_ < 10%^42,43^. As shown in Fig. 3 The temperature thresholds on determining lake ice phenology are then defined as the temperature of the date when water area percentage (*R*_*water*_) drops down below 10% or rises above 90%. The multi-year medians of the temperature thresholds are finally considered as the overall thresholds to determine the starting and ending dates of freezing-up (*T*_*FUS*_ and *T*_*FUE*_) and breaking-up of lake ice phenology (*T*_*BUS*_ and *T*_*BUE*_) separately for each lake.

### Lake ice phenology determination

The long-term lake ice phenology for the period 1978–2016 is derived from the simulated lake surface temperature given the temperature thresholds. The lake ice phenology considered herein consists of 6 indicators reflecting the timing or duration of different freezing/thawing phases. In addition to starting and ending dates of freezing-up (FUS and FUE) and breaking-up (BUS and BUE), the ice-covered duration (IceD, in days) and the fully ice-covered duration (CID, in days) are also presented in the dataset. The indicators are defined as:

6 $$\left\{\begin{array}{c}FUS={\min }\left(t\right)s.t.\,LSW{T}_{t} < {T}_{FUS}\,and\,LSW{T}_{t-1} > {T}_{FUS}\\ FUE={\max }\left(t\right)s.t.\,LSW{T}_{t} < {T}_{FUE}\,and\,LSW{T}_{t-1} > {T}_{FUE}\\ BUS={\min }\left(t\right),s.t.\,LSW{T}_{t} > {T}_{BUS}\,and\,LSW{T}_{t-1} < {T}_{BUS}\\ BUE={\max }\left(t\right),s.t.\,LSW{T}_{t} > {T}_{BUE}\,and\,LSW{T}_{t-1} < {T}_{BUE}\\ IceD=BUE-FUS\\ CID=BUS-FUE\end{array}\right.$$

Where *LSWT*_*t*_ is the simulated lake surface temperature at date *t*. The date *t* is expressed as the day of a calendar year (starting at January 1^st^). Since the lake ice phenology could spans two consecutive years, we assign herein the value of *t* to range from 1 to 730, where *t*>365 represents the date in the next consecutive calendar year.

## Data Records

The lake ice phenology data across Tibetan Plateau is archived and openly accessible via Figshare^44^ via the link: 10.6084/m9.figshare.18852338.v2. Description about the 132 lakes is shown in the lake_info.csv file, including lake ID, name, latitude, longitude, lake area, water level, depth, water volume, freezing property, and glacial replenishment. The information about the lakes is mainly collected from Records of Lakes in China^41^, HydroLAKES^35^, and Map of Glaciers and Lakes on the Tibetan Plateau and Adjoining Regions. The Phenology folder in the dataset consists of 132 CSV files. Each CSV file is named according to the lake ID presented in lake_info.csv. The columns of each CSV file are listed in Table 1.

**Table 1:** Files and formats of the dataset.

File name	Description of each column
lake_info.csv (9 columns)	Lake ID, Name, Longitude, Latitude, Area, Elevation, Depth, Volume, Glacier.
TP_LIP_Lakes132.shp	Lake boundaries collected from National Tibetan Plateau Data Center.
XXXXX_LIP.csv (7 columns)	Year
	FUS (i.e., starting date of freezing-up).
	FUE (i.e., ending date of freezing-up).
	BUS (i.e., starting date of breaking-up).
	BUE (i.e., ending date of breaking-up).
	CID (i.e., fully ice-covering duration).
	IceD (i.e., ice-covered duration).

## Technical Validation

### Quality control

The reconstructed LIP dataset is produced with strict quality control throughout the procedures. The boundaries of the lakes are examined via professional GIS tools (both ArcGIS and Google Map) to ensure the consistency of their geographic locations, and topography. Lake-wide mean surface temperature instead of that of lake centroid is derived from MOD11A2 for model calibration. The 132 lakes presented in the dataset are all with good model performance (Nash-Sutcliffe efficiency coefficient^45^ above 0.6) when the simulated lake surface temperature is compared against the MODIS-based one. Suspicious outliers (i.e., abnormal extremely high or low values) are eliminated from satellite-based lake surface temperature and snow product in providing reliable data for robust model calibration and validation. The MOD10A1 is used as an aid in determining the temperature thresholds of LIP. It is noted that the MOD10A1 is not always of good quality in continuously retrieving the ice-covered ratio of a lake due to accidental misclassification and effects of cloud cover. To alleviate the uncertainties induced by the optical RS data, we use open water ratio (see Methods) instead of directly using ice-covered ratio of a lake in determining the temperature threshold, and the medians of the temperature thresholds are finally accepted for robust lake ice phenology identification. The reconstructed LIP dataset is validated by comparing against the published LIP data as described below.

### Validation of the reconstructed LIP dataset

There is little *in-situ* observation of lake ice phenology in the TP available for a complete one-by-one validation of our reconstructed LIP dataset. We herein validate our dataset in two different ways. Firstly, we validate the effectiveness of our approach by comparing the reconstructed LIP against *in-situ* observations of two lakes in the TP^46,47^ and one lake in Canda^48^. The comparison is shown in Supplementary Fig. S1 suggesting that the estimated ice phenology overall is comparable with the *in-situ* observations, where the root mean squared error (RMSE) of the estimated FUE in Nam Co is around 7 days, RMSE of the estimated BUE in Lesser Slave Lake is near 9 days and RMSE of the estimated in Qinghai Lake is around 12 days. It should be noted that the bias could be due to both the uncertainties in the model and in the climate input to the model as well.

**Fig. 4:**
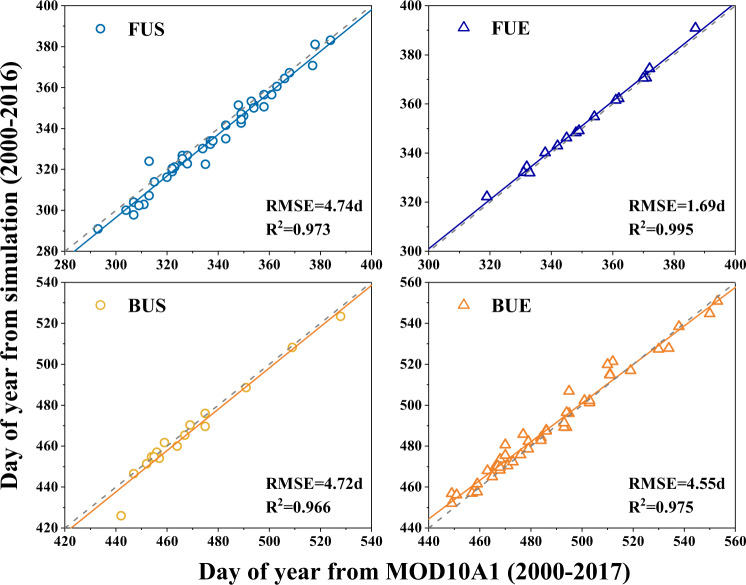
Comparison of long-term mean lake ice phenology derived in this research against that from Cai *et al*.^32^, where FUS, FUE, BUS, BUE represents starting date of freezing-up, ending date of freezing-up, starting date of breaking-up, and ending date of breaking-up respectively. The unit of FUS, FUE, BUS and BUE is day of year with the number larger than 365 indicates dates in the next calendar year.

We further compare the reconstructed LIP against two existing datasets developed based on remotely sensed data, which were published by Cai *et al*.^32^ and Qiu *et al*.^23^ respectively. The dataset published by Cai *et al*.^32^ is retrieved from MOD10A1 and Landsat data, providing mean lake ice phenology of 44 TP lakes for the period 2000–2017. Figure 4 shows the comparison of the mean annual LIP from our reconstructed dataset against that from Cai *et al*. for the 44 lakes. It can be seen that the LIP dataset produced by our approach is in high agreement with that solely based on MOD10A1, where the R^2^ for all the four phenology indices (i.e., FUS, FUE, BUS and BUE) are higher than 0.96 and the root mean squared errors (i.e., RMSE) are all within 5 days.

**Fig. 5:**
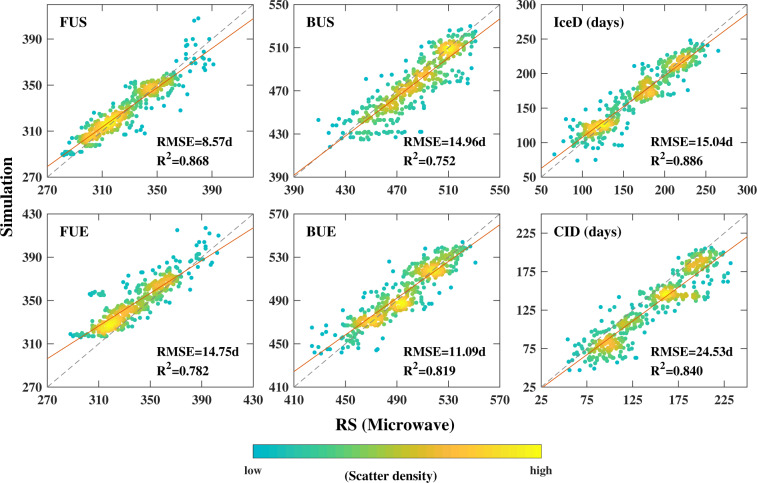
Comparison of lake ice phenology derived in this research against that from Qiu *et al*.^23^, where IceD and CID represents ice-covered duration and fully ice-covering duration respectively with unit day. The meaning and unit of FUS, FUE, BUS and BUE are the same to that in Fig. 4.

The reconstructed LIP time series is also compared against the dataset published by Qiu *et al*.^23^, which was derived from microwave brightness temperature measured by AMSR-E, AMSR2 and MWRI. The dataset from Qiu *et al*. provides LIP of 51 lakes in High Asia for the period from 2002 to 2016, among which there are 35 lakes identical to the lakes in our dataset. For the 35 lakes in the period 2002–2015 (sample size = 35 × 14), the statistically significant correlations are found between the two datasets for all the six phenology indices with R^2^ all above 0.75 (Fig. 5). The discrepancy between the two datasets is found to be the lowest in FUS (RMSE = 8.6 days) and to be the highest in BUS (RMSE = 15 days). The relative higher discrepancy of the two datasets in both BUS and FUE results at noticeable bias of the CID, which by definition equals to number of days between FUE and BUS. The uncertainties of both our reconstructed LIP dataset and those built on satellite-observations however need to be further quantified in the future particularly where *in-situ* observations becoming available.

### Spatial distribution of LIP across the TP

**Fig. 6:**
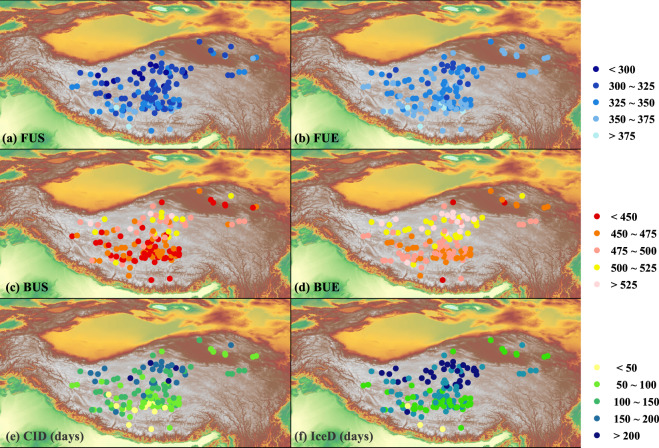
Spatial distribution of lake ice phenology over the TP. The meaning of FUS, FUE, BUS, BUE, CID and IceD are the same to that in Fig. 4 and Fig. 5.

Figure 6 shows the long-term mean spatial distribution of the LIP indices based on the reconstructed dataset. Overall, the results suggest that the phenology is close related to latitude (Fig. S2 in Supplementary). For lake in higher latitude, it tends to freeze-up earlier but break-up later hence with longer ice-covered duration. Overall, from south to north, FUS ranges from October 15^th^ to January 15^th^ while FUE varies from November 9^th^ to January 21^th^. The gradients of FUS and FUE with respect to latitude are around 6.7 days/degree and 5.7 days/degree respectively (Fig. S2 in Supplementary). For ice-breaking dates, from south to north, BUS ranges between 27^th^ January and 22^th^ June, while BUE varies between 2^nd^ March and 5^th^ July. The latitude gradients of BUS and BUE are about 11.8 days/degree and 8.5 days/degree respectively. From south to north, ice-covered duration ranges from 15 days to 215 days (CID) or from 85 to 255 days (IceD). The latitude gradients of CID and IceD are 17.5 days/degree and 15.2 days/degree respectively. Lake ice phenology is also affected by altitude (Fig. S3 in Supplementary). Statistically significant correlations are found between LIP and altitude particularly for lakes locating between 4500 to 5000 m (account for 70% of lakes in this dataset). Overall, FUS and FUE are in negative correlations with altitude, while BUS, BUE, IceD and CID are in positive correlations with altitude. It suggests that for lakes with higher altitude, earlier the FUS and FUE, later BUS and BUE and longer IceD and CID can all be expected. The altitude gradient of FUS and FUE is 9.4 days/hm and 5.7 days/hm respectively, while it is around −13 days/hm and −11.7 days/hm for BUS and BUE respectively. The altitude gradient of CID and IceD is estimated to be 18.9 days/hm and 21.4 days/hm (Fig. S3 in Supplementary).

## Supplementary information

Supplementary of Arcticle

## Data Availability

Codes in developing the lake ice phenology dataset are freely available. Codes of the modified air2water model for lake surface water temperature simulation are available at: https://github.com/Siyu1993/ModifiedModel. Codes for lake ice phenology identification are written using Matlab and are available at: https://github.com/Siyu1993/MatlabCode.git.
