# Supplementary material for: Ice phenology dataset reconstructed from remote sensing and modelling for lakes over the Tibetan Plateau
Source: Sci Data. 2022 Dec 2;9:743. doi: 10.1038/s41597-022-01863-9 (PMC9718739; doi:10.1038/s41597-022-01863-9)
Supplement: Supplementary file 1 — Supplementary of Arcticle [file 41597_2022_1863_MOESM1_ESM.pdf]

Title

Ice phenology dataset reconstructed from remote sensing and modelling for lakes over the Tibetan Plateau

Authors

Yanhong Wu[1,2], Linan Guo[3,2], Bing Zhang[1,2,4]*, Hongxing Zheng[5], Lanxin Fan[1,2,4], Haojing Chi[1,2,4], Junsheng Li[1,2,4], Shenglei Wang[1,2]

Affiliations

1. International Research Centre of Big Data for Sustainable Development Goals, Beijing 100094, China
2. Key Laboratory of Remote Sensing Science, Aerospace Information Research Institute, Chinese Academy of Sciences, Beijing 100094, China
3. Institute of Tibetan Plateau Research, Chinese Academy of Sciences, Beijing 100101, China
4. University of the Chinese Academy of Sciences, Beijing 100049, China
5. CSIRO Land and Water, Canberra, ACT 2601, Australia

corresponding author: Bing Zhang (zb@radi.ac.cn)

Supplementary

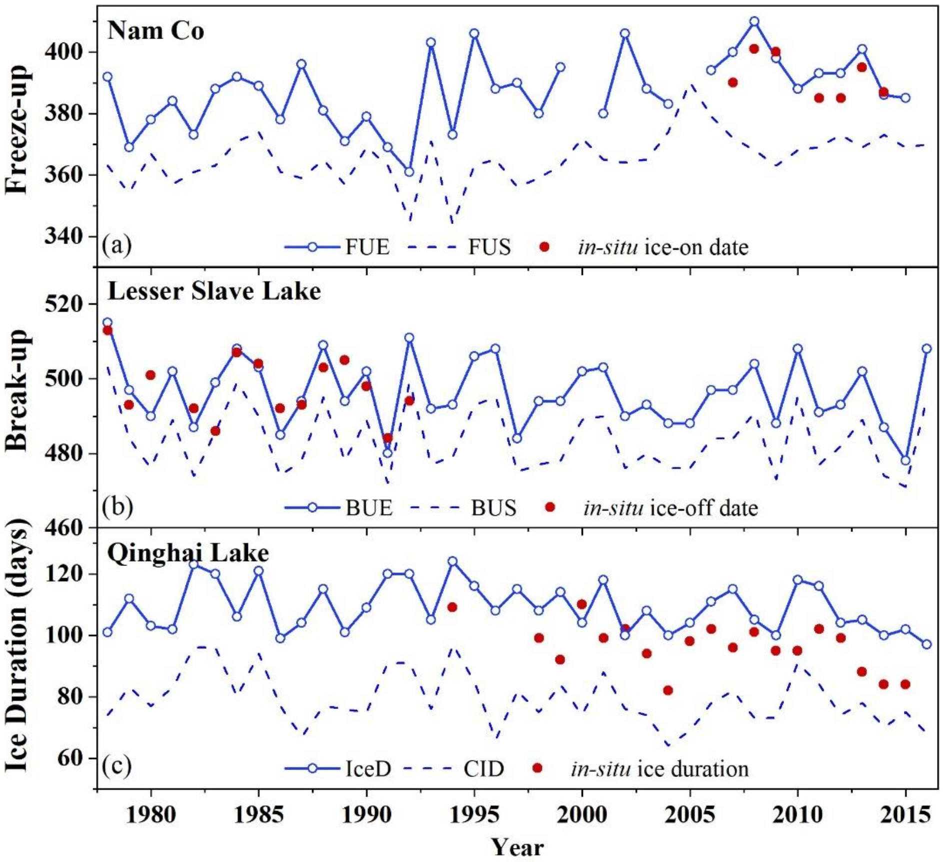

Fig. S1. Comparison of reconstructed lake ice phenology against ground-based observations. a: Nam Co with observation from Gou et al. (2017), b: Lesser Slave Lake with observation from Benson et al. (2000), c: Qinghai Lake with observation from Zhang and Duan (2021).

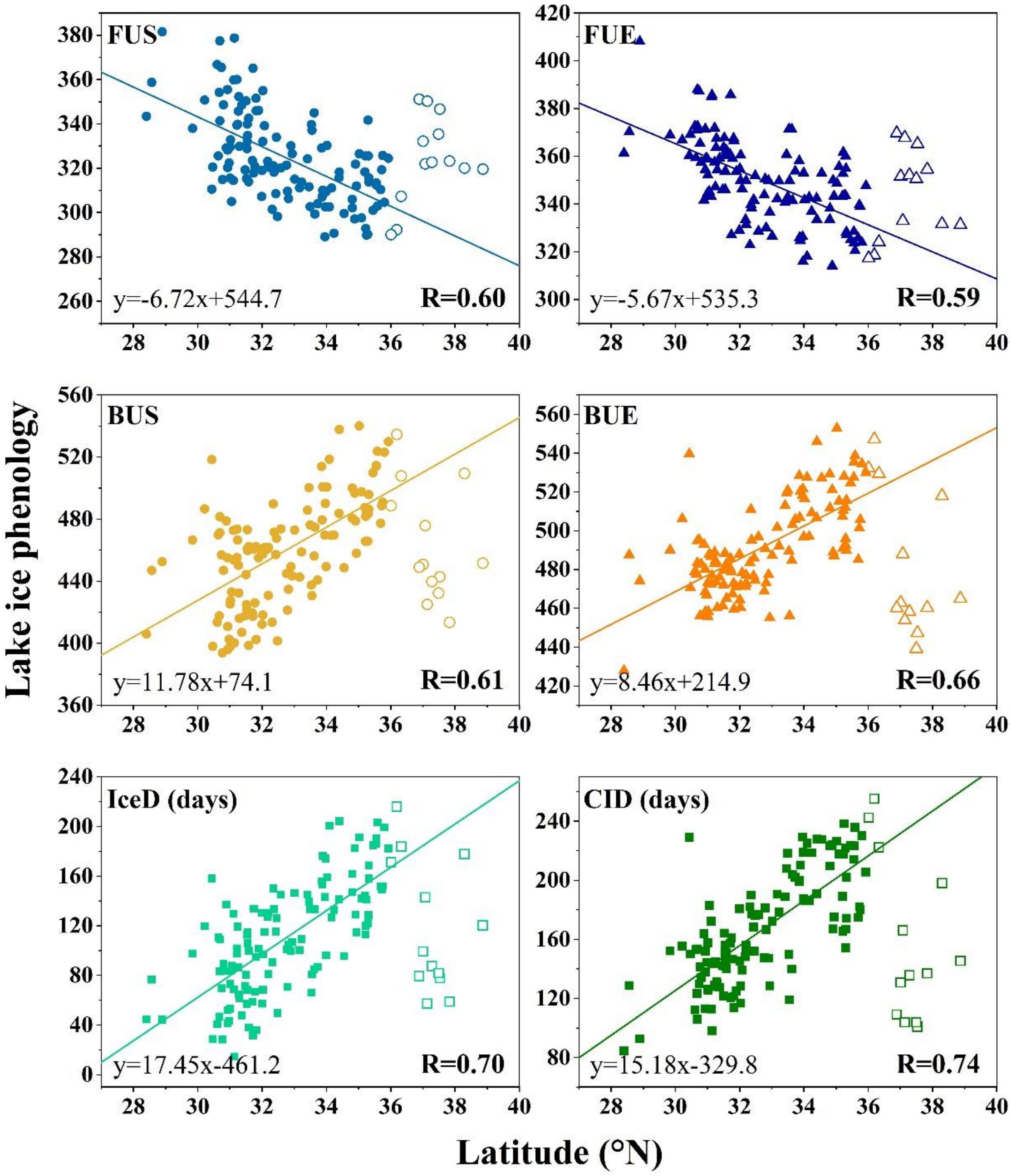

Fig. S2. Relationship between lake ice phenology and latitude. Solid points (lakes with latitude < 36°N) are used in developing the regression equations.

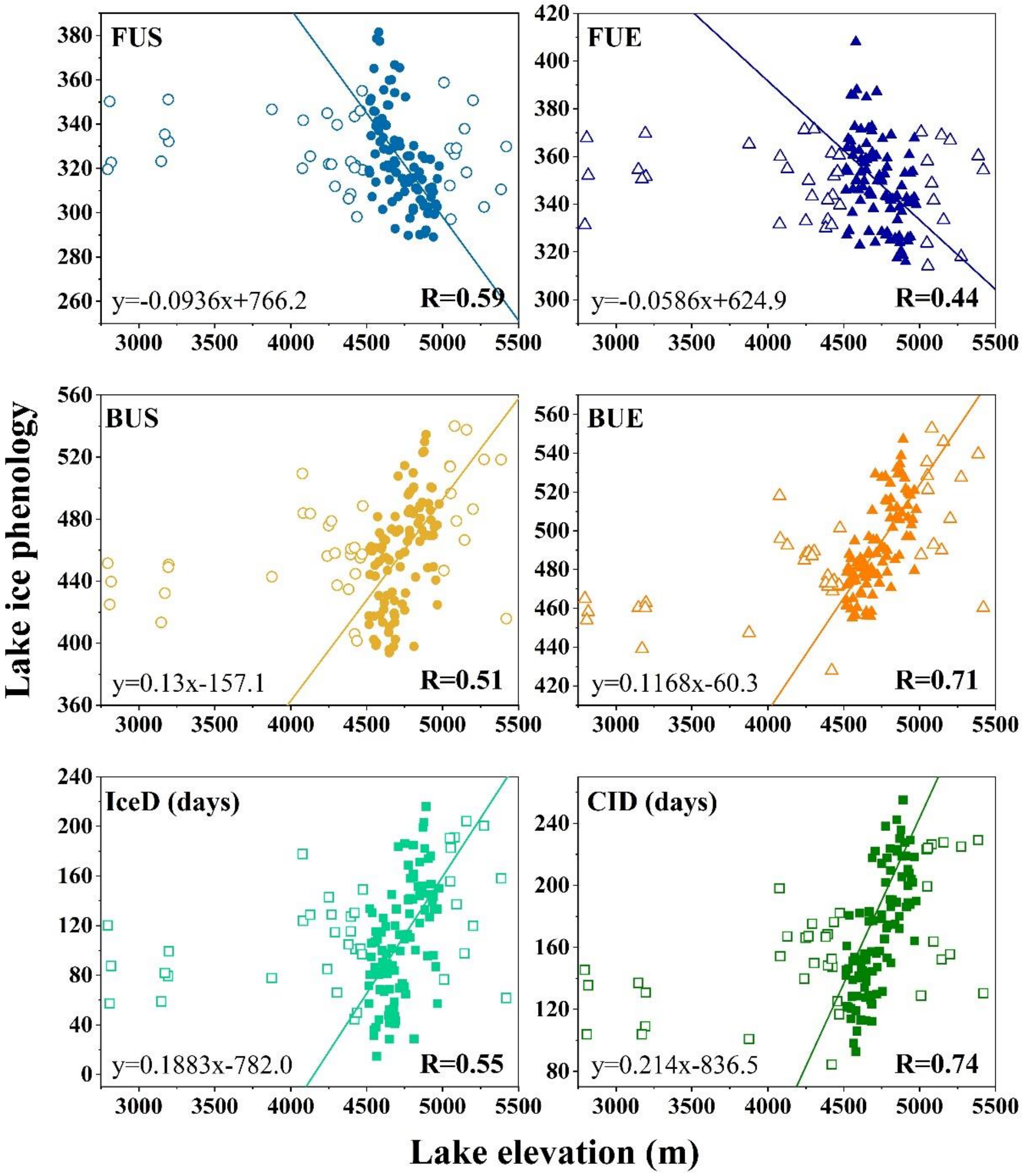

Fig. S3. Relationship between lake ice phenology and lake elevation. Solid points (lakes locate between 4500m to 5000m) are used in developing the regression equations.

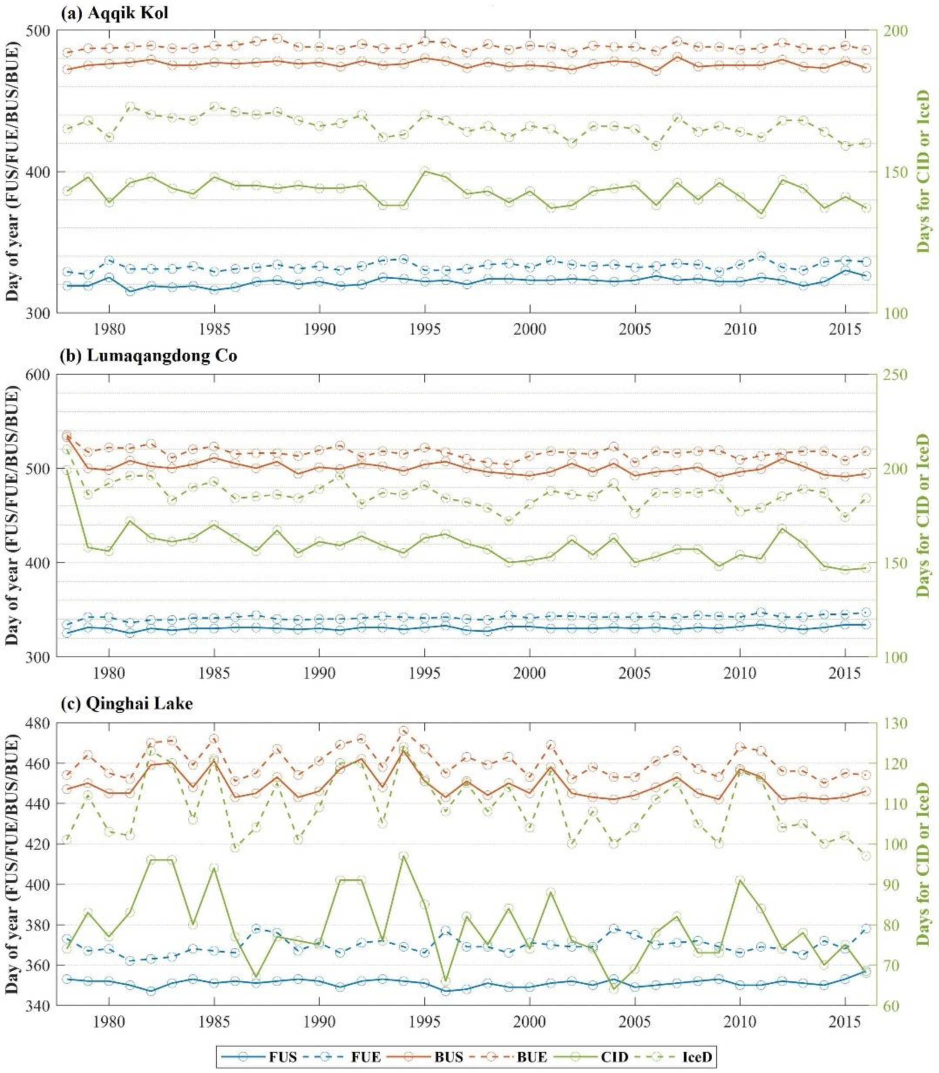

Fig. S4. Long-term variation of lake ice phenology of Aqqik Kol (a), Lumajiangdong Co (b) and Qinghai Lake (c).

References:

Benson, B., Magnuson, J., & Sharma, S. Global Lake and River Ice Phenology Database, Version 1. Boulder, Colorado USA. NSIDC: National Snow and Ice Data Center. doi: 10.7265/N5W66HP8 (2000, updated 2020).

Gou, P. *et al.* Lake ice phenology of Nam Co, Central Tibetan Plateau, China, derived from multiple MODIS data products. *Journal of Great Lakes Research* **43** (2017).

Muñoz Sabater, J. ERA5-Land hourly data from 1981 to present. Copernicus Climate Change Service (C3S) Climate Data Store (CDS). doi: 10.24381/cds.e2161bac (2019).

Zhang, G. Q. & Duan, S. Q. Lakes as sentinels of climate change on the Tibetan Plateau. *All Earth* **33**, 161-165, doi:10.1080/27669645.2021.2015870 (2021).
